# Marcador Prognóstico Eletrocardiográfico na Hipertensão Arterial Pulmonar: Tempo de RS

**DOI:** 10.36660/abc.20240083

**Published:** 2024-08-21

**Authors:** Emin Koyun, Anil Sahin, Ahmet Yilmaz, Ferhat Dindas, Idris Bugra Cerik, Gorkem Berna Koyun

**Affiliations:** 1 Sivas Numune Hospital Department of Cardiology Sivas Turquia Sivas Numune Hospital, Department of Cardiology, Sivas – Turquia; 2 Sivas Cumhuriyet University Hospital Department of Cardiology Sivas Turquia Sivas Cumhuriyet University Hospital, Department of Cardiology, Sivas – Turquia; 3 Usak University Training and Research Hospital Department of Cardiology Usak Turquia Usak University Training and Research Hospital, Department of Cardiology, Usak – Turquia; 4 Ordu University Training and Research Hospital Department of Cardiology Ordu Turquia Ordu University Training and Research Hospital, Department of Cardiology, Ordu – Turquia; 5 Sivas Cumhuriyet University Hospital Sivas Turquia Sivas Cumhuriyet University Hospital, Department of Chest Disease, Sivas – Turquia

**Keywords:** Eletrocardiografia, Prognóstico, Hipertensão Pulmonar

## Abstract

**Fundamento:**

A hipertensão pulmonar é uma condição que envolve a remodelação do ventrículo direito. A remodelação contínua também está associada ao prognóstico da doença. Durante o processo de reestruturação, alterações complexas como hipertrofia e dilatação também podem se refletir nos parâmetros eletrocardiográficos.

**Objetivos:**

Nosso estudo teve como objetivo investigar a relação entre prognóstico e parâmetros eletrocardiográficos em pacientes com hipertensão arterial pulmonar.

**Métodos:**

O estudo foi desenhado retrospectivamente e incluiu pacientes com diagnóstico de hipertensão arterial pulmonar entre 2010 e 2022. Os pacientes foram divididos em dois grupos com base no resultado de sobrevida. Vários parâmetros, incluindo parâmetros eletrocardiográficos, demográficos, ecocardiográficos, de cateter e sanguíneos, foram comparados entre os dois grupos. Um valor de p <0,05 foi considerado estatisticamente significativo.

**Resultados:**

Na análise multivariada de Cox, os parâmetros que se mostraram independentemente associados à sobrevida foram o teste de caminhada de 6 minutos, pressão média da artéria pulmonar, presença de derrame pericárdico e tempo entre o início do QRS e o pico da onda S (tempo de RS) (p<0,05 para cada). De todos os parâmetros, o tempo de RS demonstrou o melhor desempenho diagnóstico (AUC: 0,832). Na análise de sobrevida, foi encontrada correlação significativa entre o tempo de RS e a sobrevida ao utilizar o valor de corte de 59,5 ms (HR: 0,06 [0,02-0,17], p < 0,001).

**Conclusões:**

De acordo com os resultados do nosso estudo, um tempo de RS mais longo está associado a um pior prognóstico em pacientes com hipertensão arterial pulmonar. Podemos obter informações sobre o curso da doença com um parâmetro simples e não invasivo.

**Figure:**
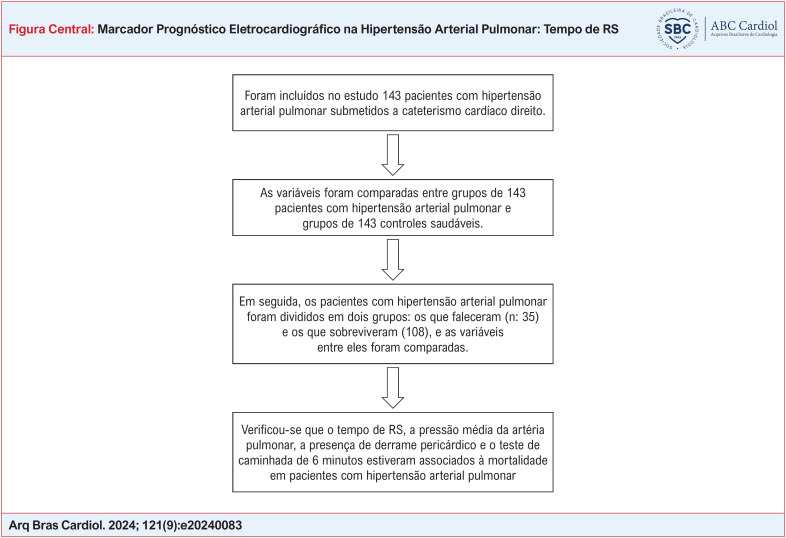


## Introdução

A hipertensão pulmonar (HP) é uma doença rara, complexa, crônica e progressiva, resultante de uma ampla variedade de condições subjacentes que direta ou indiretamente levam a pressões elevadas nas artérias pulmonares. HP é um termo hemodinâmico e fisiopatológico que abrange uma ampla gama de doenças que afetam os sistemas cardíaco e respiratório, com sintomas dependentes do sistema afetado. Sua definição hemodinâmica é a pressão média da artéria pulmonar (PMAP) medida >20 mmHg em repouso no cateterismo cardíaco direito (CCD).^1^ A hipertensão arterial pulmonar (HAP) é um grupo de HP que é diagnosticada excluindo outras causas pré-capilares, como HP tromboembólica crônica e HP por doença pulmonar. Nesse grupo, os parâmetros do CCD devem ser os seguintes: PMAP>20 mmHg, pressão capilar pulmonar (PCP) ≤15 mmHg e resistência vascular pulmonar (RVP)>2 unidades Wood (WU).^2^

O algoritmo atual de tratamento para pacientes com HAP requer avaliação frequente do paciente sobre o prognóstico e escalonamento da terapia se o status de baixo risco não for alcançado. Foram propostos exame físico para avaliação de risco, teste de exercício cardiopulmonar, classe funcional da Organização Mundial da Saúde (CF-OMS), nível de peptídeo natriurético tipo pró-cérebro N-terminal (NT-proBNP), teste de caminhada de 6 minutos (TC6), exames de imagem e vários parâmetros diagnósticos, incluindo CCD.^3^

Até o momento, existem estudos limitados sobre o papel do eletrocardiograma (ECG) no monitoramento de pacientes com HAP. Na HAP, alterações como dilatação e hipertrofia ocorrem no ventrículo direito (VD) ao longo do tempo devido à pressão elevada da artéria pulmonar. Estas alterações também podem ser refletidas nos ECGs de superfície dos pacientes. Embora tenha sido demonstrado que parâmetros hemodinâmicos e hipertrofia ventricular direita causam alterações significativas no ECG,^4^ seu valor prognóstico não foi extensivamente avaliado.

Em um estudo recente sobre embolia pulmonar aguda, doença que afeta o coração direito, constatou-se que o tempo entre o início do QRS e o pico da onda S (tempo de RS) está associado à mortalidade.^5^ Entretanto, não foi investigado se o tempo de RS está relacionado à mortalidade em subgrupos específicos de HP, especialmente em condições crônicas. Ainda não está claro se os parâmetros do ECG no momento do diagnóstico fornecem informações sobre o curso da doença e a sobrevida em pacientes com HP. Na HAP ocorre atraso no sistema de condução intracardíaca, principalmente pelo aumento da pressão nas câmaras cardíacas direitas. Como resultado do atraso na condução elétrica, ocorre estiramento na onda S.^5^ Como consequência, faz com que o tempo de RS aumente.^5^ Portanto, nosso objetivo foi investigar a relação entre os padrões de ECG de pacientes com HAP no momento do diagnóstico, especialmente a duração do RS, e o prognóstico da doença.

## Métodos

Todos os pacientes submetidos a um CCD em hospital terciário entre 2010 e 2022 e com diagnóstico de HAP do grupo 1 foram incluídos no estudo retrospectivo. O número total de pacientes com HAP que atenderam a esses critérios foi determinado como 143. Em seguida, por conveniência, 143 voluntários saudáveis, pareados em termos de idade e sexo e iguais ao número do grupo de pacientes, foram incluídos no estudo como um grupo de controle. Na primeira etapa, foram comparadas características demográficas, ECG, parâmetros sanguíneos e parâmetros ecocardiográficos entre pacientes com diagnóstico de HAP e o grupo controle de voluntários saudáveis. Em seguida, os pacientes com HAP foram divididos em dois grupos: vivos e mortos. Características demográficas, ECG, CCD, ecocardiografia e parâmetros sanguíneos foram comparados entre pacientes sobreviventes e falecidos (Figura Central).

Todos os pacientes com HAP incluídos no estudo apresentavam risco médio ou alto para HP na ecocardiografia transtorácica. Portanto, esses pacientes foram submetidos ao CCD. Os critérios de inclusão para o estudo são os seguintes: diagnóstico de HAP do grupo 1 de acordo com a classificação clínica da diretriz de HP criada pela *European Cardiology* e *European Respiratory Society* em 2022; CCD e PMAP>20 mmHg, PCP≤15, RVP>2 WU; ser maior de 18 anos; presença de ECG no momento do diagnóstico.

Os critérios de exclusão do estudo são os seguintes: ter diagnóstico de HP por cardiopatia esquerda; diagnóstico de HP associada a doenças pulmonares e hipóxia; diagnóstico de HP por oclusões de artérias pulmonares; ter diagnóstico de DP com mecanismos pouco claros e multifatoriais; ser menor de 18 anos; falta de dados sobre os parâmetros CCD no momento do diagnóstico; ausência de ECG ao diagnóstico; ter insuficiência renal ou hepática grave; ter histórico de malignidade.

**Figura 1:**
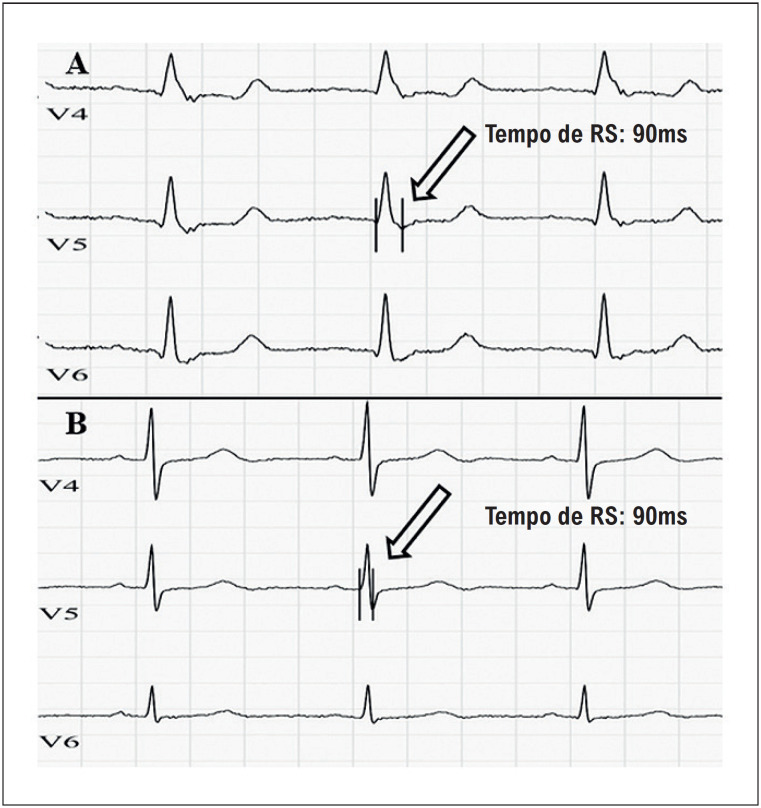
Cálculo do tempo de RS nos eletrocardiogramas dos pacientes. Exemplos de eletrocardiogramas mostrando tempo de RS aumentado (A) e tempo de RS normal (B).

Os ECGs dos pacientes no momento do diagnóstico foram encontrados nos prontuários dos pacientes. ECGs realizados quando os pacientes foram diagnosticados com HP foram incluídos no estudo. ECGs com 12 derivações, velocidade do papel de 25 mm/s e 10 mm/mV foram digitalizados e carregados no software do computador, e todas as medidas foram feitas com o software do computador. Dois cardiologistas realizaram os exames de ECG utilizando este software. Caso fossem detectados problemas nos exames de ECG, era recebido apoio de um terceiro cardiologista. Os cardiologistas que revisaram os ECGs desconheciam os resultados dos pacientes. No ECG, a arritmia atrial, tempo QT, tempo PR, inversão da onda T, duração do QRS, desvio do eixo direito, depressão ST, tempo de RS e frequência cardíaca por minuto foram analisados em ambiente computacional. O tempo QRS foi medido a partir da derivação onde o tempo entre o início da onda QRS e o ponto J foi maior. Em pacientes com HP, ocorre atraso na condução e orientação direita e posterior do vetor QRS devido a alterações hemodinâmicas. Consequentemente, ocorre um prolongamento da duração do RS nas derivações inferolaterais do ECG. Portanto, calculamos a duração do RS no ECG dos pacientes a partir das derivações inferolaterais. O tempo de RS foi calculado a partir da derivação com maior tempo de RS entre as derivações inferolaterais (D1, D2, D3, AVL, AVF, V4, V5 e V6).^5^ O tempo entre o ponto inicial da onda QRS e o ponto mais baixo da onda S ou S’ foi determinado como o tempo de RS^5^ (Figura 1). A unidade de medida foi determinada em milissegundos.

### Análise estatística

O teste de Kolmogorov-Smirnov foi utilizado para determinar a distribuição normal dos dados. O teste U de Mann-Whitney ou o teste-t de Student não pareado foram utilizados para detectar diferenças nos dados de variáveis contínuas. Os dados contínuos foram expressos como média ± desvio padrão (DP) ou mediana e intervalo interquartil. As variáveis categóricas foram expressas como frequências absolutas (n) e relativas (%) A análise do qui-quadrado foi utilizada para comparar as variáveis categóricas. Análise de regressão univariada e multivariada de Cox foi realizada para determinar parâmetros preditivos de mortalidade. Variáveis estatisticamente significativas (p<0,05) na análise de regressão univariada de Cox foram incluídas na análise de regressão multivariada de Cox. A análise da curva *Receiver Operating Characteristic* (ROC) foi realizada para obter o valor de corte e a área sob a curva (AUC) dos parâmetros preditores de mortalidade. Utilizando a análise de correlação de Pearson, foi examinada a relação entre o tempo de RS e as funções cardíacas direitas na ecocardiografia. A análise de Kaplan-Meier foi realizada para examinar a relação entre o tempo de RS, que apresenta o maior desempenho diagnóstico de acordo com a análise da curva ROC, e a sobrevida. Os dados foram analisados com o programa estatístico SPSS 22.0. Afirmou-se que era necessário p<0,05 para que os dados fossem considerados estatisticamente significativos.

## Resultados

Quando foram comparados parâmetros demográficos, laboratoriais, ecocardiográficos e eletrocardiográficos da HAP (n=143) e do grupo controle (n=143), foram observadas duração do RS, duração do QRS, desvio do eixo para a direita, arritmia atrial, infradesnivelamento do segmento ST e a inversão da onda T a qual foi maior no grupo HAP do que no grupo controle e foi estatisticamente significativa. Uma comparação detalhada dos parâmetros entre os grupos é mostrada na Tabela 1.

**Tabela 1:** Comparação das variáveis entre o grupo hipertensão arterial pulmonar e o grupo controle

Variáveis		Grupo HAP(n=143)	Grupo de controle(n=143)	p-valor
Idade (anos)		62,32±13,43	59,79±14,45	0,12
Masculino n (%)		36 (25,17)	42 (29,37)	0,42
Índice de massa corporal (kg/m^2^)		28,74±1,81	26,53± 1,74	0,09
Parâmetros ecocardiográficos				
	Fração de ejeção do ventrículo esquerdo (%)	55,15±3,26	56,49±3,13	0,37
	Diâmetro do átrio esquerdo (mm)	40,1±5,3	36,4±3,3	0,08
	Septo interventricular (mm)	10,4±1,3	10,1±1,4	0,13
Parâmetros sanguíneos				
	Hemoglobina (g/dL)	13,7±2,1	14±1,9	0,29
	Glóbulo branco (10^3^ /uL)	8,1±2,87	8,3±2,89	0,52
	Plaquetas (10^3^ /uL)	231,3±70,1	240,8±76,9	0,27
	Sódio (mEq/L)	137,8±3,7	138,2±3,2	0,31
	Potássio (mEq/L)	4,37±0,47	4,42±0,48	0,43
	Cálcio (mg/dL)	8,92±0,55	8,89±0,55	0,73
	Creatinina (mg/dL)	0,83±0,24	0,80±0,19	0,29
	Aspartato aminotransferase (UI/L)	21,2±11,3	22,2±10,4	0,45
	Alanina aminotransferase (UI/L)	18±15,5	21,2±19	0,12
Parâmetros de ECG				
	Tempo QT (ms)	402±5	403±12	0,14
	Tempo PR (ms)	142,7±16	143,4±11,5	0,66
	Frequência cardíaca por minuto	82,8±14,5	81±15,3	0,36
	Tempo de RS (ms)	60,7±10,2	55±4,3	<0,001
	Tempo QRS (ms)	100,2±14,6	96,8±13,6	0,04
	Desvio do eixo direito, n (%)	22 (15,38)	7 (4,8)	0,002
	Depressão ST, n (%)	25 (17,4)	3 (2,09)	<0,001
	Arritmia atrial, n (%)	27 (18,88)	0 (0)	<0,001
	Inversão da onda T, n (%)	21 (14,68)	6 (4,19)	<0,001

Os dados são expressos como n (%), média ± desvio padrão. HAP: hipertensão arterial pulmonar; ECG: eletrocardiograma.

Os pacientes com HAP foram divididos em dois grupos: pacientes sobreviventes (n=108) e pacientes falecidos (n=35). Características demográficas, comorbidades, tratamentos utilizados, TC6, classificação CF-OMS, parâmetros laboratoriais, ecocardiográficos, ECG e CCD foram comparados entre os dois grupos. No grupo de pacientes falecidos, o TC6M foi menor e considerado estatisticamente significativo. CF-OMS 3-4, PMAP, derrame pericárdico, velocidade do jato tricúspide, tempo de RS e tempo QRS foram maiores no grupo falecido e foram considerados estatisticamente significativos. A comparação detalhada entre os grupos de pacientes falecidos e sobreviventes em pacientes com HAP é mostrada na Tabela 2.

**Tabela 2:** Comparação dos parâmetros demográficos, ecocardiográficos, eletrocardiográficos, cateterismo cardíaco direito e laboratoriais de pacientes com hipertensão arterial pulmonar falecidos e sobreviventes

Variáveis		Morte(35)	Sobrevivente(108)	p-valor
Idade (anos)		64,45±10,6	61,63±14,1	0,21
Masculino, n (%)		10 (28,57)	26(24,07)	0,59
Índice de massa corporal (kg/m^2^)		28,4±1,9	28,8±1,7	0,24
Teste de caminhada de 6 minutos (m)		210,8±92,4	261,5±97,3	0,012
CF-OMS 3-4, n (%)		14 (40)	25 (23,14)	0,036
Diabetes mellitus, n (%)		7 (20)	21(19,44)	0,9
Hipertensão, n (%)		9 (25,7)	28(25,92)	0,9
Doença arterial coronariana, n (%)		3 (8,57)	5(4,62)	0,38
**Parâmetros de CCD**				
	PSAP (mmHg)	64,3±17,2	55,5±15,1	0,06
	PAAP (mmHg)	34,4±9,3	26,2±7,6	0,09
	PMAP (mmHg)	44,2±15,8	36,1±11,4	0,007
	Qp/Qs	1,04±0,20	1,04±0,13	0,82
	Vasorreatividade, n (%)	2 (5,7)	7 (6,5)	0,87
	RVP (WU)	7,05±3,2	7,18±2,6	0,80
	RVS (WU)	20.1±7	20,5±5	0,68
	RVP/RVS	0,357±0,09	0,359±0,11	0,90
	Índice cardíaco (L/min/m^2^)	2,79±0,44	2,81±0,46	0,81
	Pressão parcial de oxigênio (%)	48,59±9,1	48,22±8,9	0,83
	PAD (mmHg)	8,70±1,32	8,76±1,71	0,84
	Débito cardíaco (L/min)	4,75±0,78	4,63±0,83	0,46
	PCP (mmHg)	11.2±3,1	10,5±2,9	0,24
	Pressão sistólica aórtica (mmHg)	114,9±9,8	117,5±10,1	0,19
	Pressão diastólica aórtica (mmHg)	65,2±12,7	69,1±12,3	0,11
**Parâmetros ecocardiográficos**				
	Fração de ejeção do ventrículo esquerdo (%)	54,7±3,27	55,2±3,26	0,36
	Diâmetro do átrio esquerdo (cm)	4,07±0,64	3,99±0,48	0,50
	Septo interventricular (cm)	1,06±0,17	1,03±0,12	0,31
	Dilatação do ventrículo direito, n (%)	20 (57,14)	55 (50,92)	0,19
	Derrame pericárdico, n (%)	13 (37,14)	5 (4,62)	<0,001
	Velocidade do jato tricúspide (m/s)	3,96(3,6-4,2)	3,72(3,4-4)	0,037
	PSAP (mmHg)	69±16,7	61±18,8	0,07
**Parâmetros laboratoriais**				
	Hemoglobina (g/dL)	13,9±2,3	13.6±2,0	0,56
	Plaquetas (10^3^ /uL)	229,1±91,7	232±62,11	0,83
	Sódio (mEq/L)	137,2±3,06	138±3,97	0,28
	Potássio (mEq/L)	4,43±0,55	4,36±0,44	0,45
	Cálcio (mg/dL)	8,84±0,60	8,94±0,53	0,33
	Creatinina (mg/dL)	0,87±0,22	0,81±0,40	0,26
	NT-proBNP (pg/ml)	1491(0-2542)	1261(0-2180)	0,33
	Lipoproteína de alta densidade (mg/dL)	39,9±14,2	43±12,1	0,27
	Lipoproteína de baixa densidade (mg/dL)	110,6±30,3	102,6±36,8	0,26
	Triglicerídeos (mg/dL)	134,5±58,6	129,6±64,8	0,70
	Aspartato aminotransferase (UI/L)	19,4±16,6	21,8±9	0,42
	Alanina aminotransferase (UI/L)	17,3±15,4	18,2±15,6	0,77
**Medicamentos**				
	Inibidor da enzima conversora de angiotensina, n (%)	10(28,57)	29 (26,85)	0,95
	Bloqueador dos canais de cálcio, n (%)	7(20)	21 (19,44)	0,23
	Betabloqueador, n (%)	9(25,71)	23 (21,29)	0,58
	Antiagregante, n (%)	2(5,71)	6 (5,55)	0,52
	Anticoagulante, n (%)	9(25,71)	21 (19,44)	0,43
	Estatina, n (%)	8(22,85)	25 (23,14)	0,26
	Antagonista do receptor de endotelina, n (%)	17(48,57)	59 (54,62)	0,50
	Inibidores PDEi-5, n (%)	3(8,57)	10 (9,25)	0,51
	Riosiguat, n (%)	2(5,71)	8 (7,76)	0,46
	Prostanoid, n (%)	6(17,14)	19(17,59)	0,65
**Parâmetros eletrocardiográficos**				
	Tempo QT (ms)	401,8±5,4	402,1±4,9	0,74
	Tempo PR (ms)	141±16	143,2±16	0,49
	Frequência cardíaca por minuto	84,3±15,4	82,2±142	0,47
	Tempo de RS (ms)	71,6±12,2	57.1±6,2	<0,001
	Tempo QRS (ms)	107±12,8	98±14,4	0,001
	Desvio do eixo direito, n (%)	5(14,28)	17(15,74)	0,38
	Depressão ST, n (%)	6(17,14)	19(17,59)	0,96
	Arritmia atrial, n (%)	7(20)	20(18,51)	0,85
	Inversão da onda T, n (%)	5(14,28)	16(14,81)	0,92

Os dados estão expressos em n (%), média ± desvio padrão e mediana (1º quartil - 3º quartil). PSAP: pressão sistólica da artéria pulmonar; PMAP: pressão média da artéria pulmonar; NT-proBNP: peptídeo natriurético tipo pró B terminal; PCP: pressão de oclusão capilar pulmonar; PDEi-5: fosfodiesterase tipo 5; RVP: resistência vascular pulmonar; PAD: pressão de átrio direito; RVS: resistência vascular sistêmica; CF-OMS: classificação funcional da Organização Mundial da Saúde; CCD: cateterismo cardíaco direito.

Análise de regressão univariada e multivariada de Cox foi realizada para identificar preditores de mortalidade em pacientes com HAP. Na análise de regressão multivariada de Cox, o TC6, a PMAP, a presença de derrame pericárdico e o tempo de RS foram considerados preditores independentes de mortalidade em pacientes com HAP (Tabela 3).

**Tabela 3:** Análise de regressão de Cox univariada e multivariada para identificação de preditores de mortalidade

Variáveis	Análise de regressão univariada				Análise de regressão multivariada			
	RH	IC		p	RH	IC		p
Teste de caminhada de 6 minutos	0,995	0,990	0,999	0,006	0,991	0,985	0,997	0,003
CF-OMS 3-4	2.492	1.247	4.979	0,013				
PMAP	1.035	1.017	1.053	<0,001	1.059	1.029	1.090	<0,001
Derrame pericárdico	4.878	2.438	9.761	<0,001	3.414	1.309	8.900	0,012
Velocidade do jato tricúspide	2.679	0,993	7.225	0,052				
Tempo de RS	1.182	1.139	1.228	<0,001	1.215	1.140	1.295	<0,001
Tempo QRS	1.038	1.017	1.059	<0,001				

IC: intervalo de confiança; HR: taxa de risco; PMAP: pressão média da artéria pulmonar; CF-OMS: classificação funcional da Organização Mundial da Saúde.

**Figura 2:**
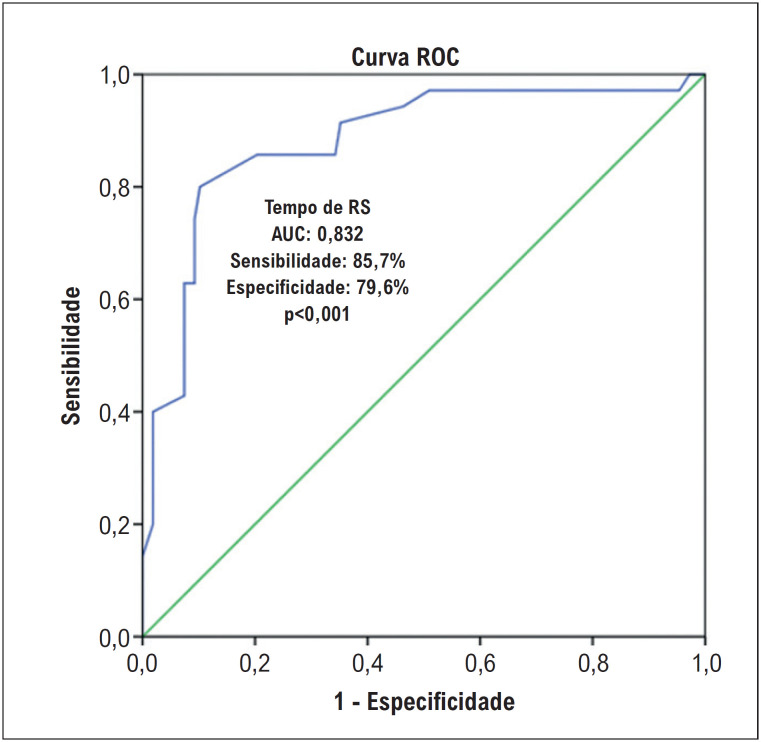
Avaliação do tempo de RS com análise ROC.

A análise ROC foi realizada para avaliar o desempenho diagnóstico do tempo de RS, que é um preditor independente de mortalidade, no prognóstico. Na análise ROC foi determinado o valor de 59,5 ms como ponto de corte para o tempo de RS. Uma duração de RS superior a 59,5 ms foi determinada para prever mortalidade em pacientes com HAP com sensibilidade de 85,7% e especificidade de 79,6% (Figura 2). De acordo com a análise da curva ROC, o valor de corte de um tempo de RS de alta sensibilidade que pode ter uso clínico pode ser determinado como 56,5 ms (sensibilidade 94%, especificidade 54%), e o valor de corte de um tempo de RS de alta especificidade pode ser determinado como 76,5 ms (sensibilidade 44%, especificidade 93%). De acordo com os resultados da análise da curva ROC, descobrimos que o parâmetro mais forte preditor de mortalidade foi o tempo de RS [(TC6; AUC: 0,658, p=0,008), (PMAP; AUC: 0,674, p=0,004), (derrame pericárdico; AUC: 0,641, p=0,019)].

Utilizando a análise de correlação de Pearson, foi examinada a relação entre o tempo de RS e as funções cardíacas direitas na ecocardiografia. De acordo com os resultados da análise, foi encontrada uma relação significativa e positiva entre o tempo de RS e a dilatação do VD (r=0,243, p<0,05), pressão sistólica da artéria pulmonar (r=0,265, p<0,05) e velocidade do jato tricúspide (r=0,652, p<0,05).

**Figura 3:**
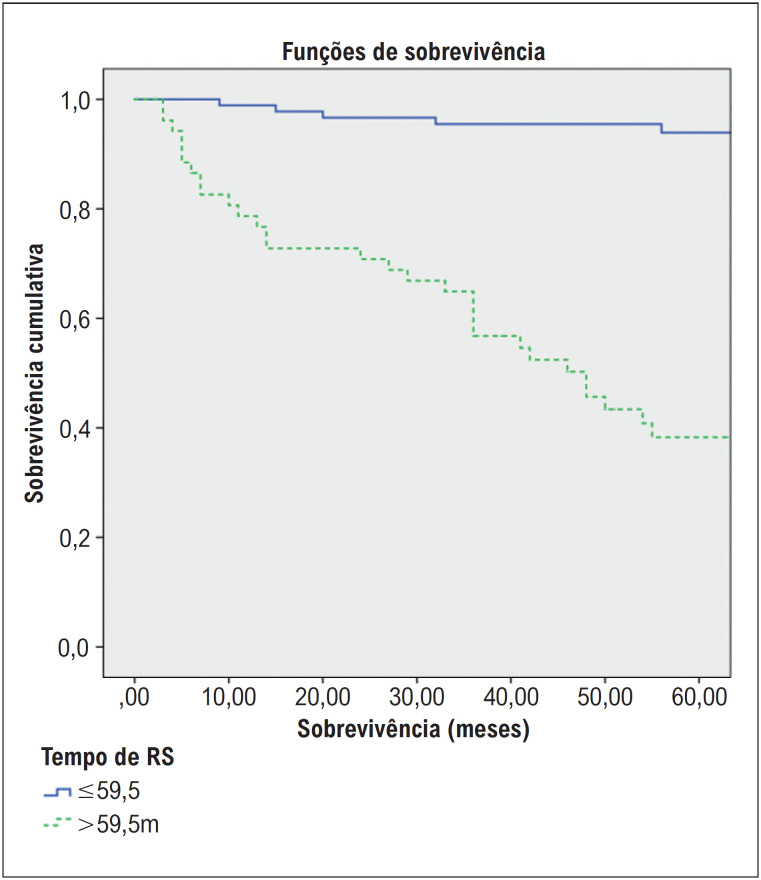
Relação entre tempo de RS e sobrevida na análise de Kaplan Meier.

O período médio de acompanhamento do grupo de pacientes falecidos após o diagnóstico de HAP foi determinado como 30,4 meses. Na análise de sobrevida de Kaplan Meier realizada definindo o valor de corte para o tempo de RS como 59,5 ms, foi encontrada uma correlação estatisticamente significativa entre o tempo de RS e a sobrevida [HR: 0,06(0,02-0,17), p<0,001)] (Figura 3).

## Discussão

De acordo com os resultados do nosso estudo, vários parâmetros podem trazer informações sobre o prognóstico dos pacientes com HAP no momento do diagnóstico. Além dos parâmetros predefinidos, o tempo de RS que podemos detectar no ECG pode nos fornecer informações importantes. Além disso, nosso estudo mostrou que pode dar melhores informações prognósticas do que muitos parâmetros avaliados no momento do diagnóstico.

A HAP é uma doença caracterizada por um aumento persistente e anormal da pressão arterial pulmonar. Como resultado, desenvolve-se insuficiência ventricular direita, com sintomas clínicos de falta de ar, fadiga, fraqueza, angina e síncope.^6^ Hoje, a HAP continua a ser uma doença crônica cuja patogênese não está totalmente elucidada.^7^ A avaliação clínica detalhada do paciente desempenha um papel fundamental na seleção do tratamento e na observação da resposta do paciente ao tratamento.^8^ As decisões terapêuticas na HP devem basear-se em parâmetros com valor prognóstico comprovado.^9^

Embora a ecocardiografia seja prioridade na prática rotineira no rastreamento da HP, o ECG é um exame recomendado para ser utilizado nas etapas diagnósticas. Contudo, apesar de uma sensibilidade e especificidade relativamente baixas, o ECG ainda é útil na fase inicial do diagnóstico da HP. Num estudo baseado em ECG realizado em crianças em idade escolar japonesas, descobriu-se que o ECG poderia prever o diagnóstico precoce de HAP.^10^ Foi demonstrado que o ECG utilizado para rastrear HP pode proporcionar benefícios significativos quando combinado com outros testes não invasivos.^11^

Várias alterações no ECG, como aumento da duração e amplitude da onda P em D2, alterações na voltagem e frequência cardíaca das derivações precordiais, alterações na duração do QTc e do QRS e a presença da onda qR na derivação V1 afetam o prognóstico em pacientes com HAP.^12^ Tonelli et al. compararam ECGs de pacientes com HAP no momento do diagnóstico e no estágio final da doença. Neste estudo, foi observado aumento na duração do QRS, duração do PR, duração do QTc, relação amplitude R/S e frequência cardíaca na derivação V1 nos ECGs realizados no último estágio da doença. Além disso, ondas T negativas nas derivações inferiores, desvio para a direita no eixo do complexo QRS e bloqueio de ramo direito foram observados com maior frequência.^13^ Em nosso estudo, a duração do QRS foi maior e a inversão da onda T foi detectada com maior frequência no grupo de pacientes falecidos.

Existem vários estudos sobre o tempo de RS. Em um estudo retrospectivo que examinou os parâmetros do ECG medidos durante o diagnóstico de embolia pulmonar aguda, foram examinadas a duração do RS e a mortalidade em um mês dos pacientes. Descobriu-se que a mortalidade dos pacientes em um mês está relacionada à maior duração do RS.^5^ No entanto, embora este estudo tenha examinado a relação entre a duração do RS e a mortalidade em curto prazo, nosso estudo examinou a relação entre a duração do RS e a mortalidade em longo prazo.

Em nosso estudo, o tempo de RS, um parâmetro eletrocardiográfico, foi maior no grupo de pacientes que faleceram durante o acompanhamento do que nos sobreviventes. Foi determinado que poderia prever a mortalidade em análises de regressão univariada e multivariada de Cox. Além disso, de acordo com nossa revisão de literatura, nosso estudo é o primeiro e maior estudo que examina a relação entre HAP e tempo de RS. As diretrizes atuais recomendam a estratificação de risco dos pacientes com HAP no início do estudo e em cada consulta de acompanhamento. Essa classificação de risco inclui diversos parâmetros, incluindo métodos clínicos, laboratoriais e de imagem. Além dos parâmetros rotineiros de estratificação de risco, a medição da duração da RS no ECG no momento do diagnóstico e durante o acompanhamento pode fortalecer as informações prognósticas.

As condições que afetam o complexo QRS também afetam a duração do RS porque o tempo de RS faz parte do tempo QRS. Na HAP, a onda QRS mais longa que o normal está associada a alterações hemodinâmicas. O aumento da pós-carga do VD causa dilatação e perda de função no VD. Esta situação pode afetar o ramo direito e as fibras de Purkinje, causando atraso ou bloqueio de condução.^14^ Na HAP, o atraso na condução e a orientação direita e posterior do vetor QRS causam prolongamento da duração do RS, especialmente nas derivações ínfero-laterais. Em pessoas sem doença cardíaca, a onda S nas derivações V4-V5-V6 é causada pela direção das forças elétricas do VD e septal em direção à base do coração e das forças elétricas do ventrículo esquerdo em direção à parte posterior do coração.^15^ Portanto, o tempo de RS nas derivações inferolaterais pode ter previsto um pior prognóstico na HAP melhor do que outros parâmetros do ECG.

A ecocardiografia tem um papel muito importante no diagnóstico da HAP. Isso ocorre porque está prontamente disponível e não é invasiva. Além disso, muitos parâmetros medidos pela ecocardiografia demonstraram estar relacionados à hemodinâmica pulmonar.^16^ Portanto, a ecocardiografia pode nos fornecer parâmetros hemodinâmicos importantes para o diagnóstico, acompanhamento e prognóstico de pacientes com HAP.^17^ Em pacientes com HAP, o aumento da pressão arterial pulmonar causa hipertrofia e dilatação do VD.^18,19^ Como resultado, o VD aumenta e pode tornar-se maior que o ventrículo esquerdo ao longo do tempo. Portanto, mais dilatação ventricular direita e pior evolução clínica foram detectadas em pacientes com HAP.^20^ Após alterações no VD, pode ocorrer insuficiência ventricular direita nestes pacientes. Como resultado da insuficiência ventricular direita, pode começar a insuficiência da valva tricúspide e pode ocorrer um aumento na velocidade do jato na valva tricúspide. É importante avaliar ecocardiograficamente o derrame pericárdico em pacientes com HAP. Estudos constataram que pacientes com HAP e derrame pericárdico apresentam desfechos clínicos ruins e alta taxa de mortalidade.^21^ Quando os parâmetros ecocardiográficos foram comparados entre pacientes falecidos e vivos com HAP, observou-se que a velocidade do jato tricúspide e a taxa de derrame pericárdico foram maiores no grupo de pacientes falecidos. Porém, de acordo com os resultados da análise de regressão, constatou-se que houve relação significativa apenas entre derrame pericárdico e mortalidade entre os parâmetros ecocardiográficos. Isto foi considerado consistente com a literatura atual. Portanto, a avaliação regular da presença de derrame pericárdico em pacientes com HAP durante o período de acompanhamento é muito importante para o seu benefício prognóstico.

A capacidade de exercício está associada à sobrevida e ao estado funcional em pacientes com HP.^22^ A capacidade de exercício avaliada pelo teste de caminhada de seis minutos tem sido um parâmetro obrigatório nos estudos clínicos mais recentes sobre HAP.^23^ Além disso, um estudo descobriu que o TC6 também poderia avaliar a dessaturação de oxigênio induzida pelo exercício em pacientes com doença vascular pulmonar.^24^ No entanto, poucos estudos foram realizados para investigar a relação entre a função pulmonar e a dessaturação relacionada ao esforço em pacientes com HP.^25^ Em nosso estudo, o TC6 médio foi encontrado ser menor em pacientes falecidos com HAP do que em sobreviventes. Além disso, análises de regressão univariada e multivariada de Cox concluíram que havia uma relação significativa entre mortalidade e TC6. Nessa perspectiva, acredita-se que procurar aumentar o TC6 no acompanhamento e tratamento de pacientes com HAP seja uma meta de tratamento correta.

A PMAP é um parâmetro importante no diagnóstico da doença.^26^ Em nosso estudo, a PMAP medida pelo CCD foi estatisticamente significativamente maior no grupo de pacientes falecidos. Além disso, análises de regressão revelaram associação significativa entre PMAP e mortalidade. Embora as diretrizes atuais não aceitem a PMAP medida pelo CCD como parâmetro de risco no acompanhamento do tratamento de pacientes com HAP, nossos resultados sugerem que direcionar a diminuição da PMAP pode ser importante no acompanhamento da doença.

### Limitações

As principais limitações do estudo são sua natureza retrospectiva, desenho unicêntrico e pequeno número de participantes. Além disso, não incluir no estudo grupos de HP que não sejam pacientes com HAP do grupo 1 é uma limitação importante. A impossibilidade de realizar CCD e teste de esforço cardiopulmonar durante o acompanhamento também pode se mostrar como limitação.

## Conclusões

Os resultados obtidos em nosso estudo mostram que existe uma forte relação entre a duração do RS e a mortalidade em pacientes com HAP. A novidade que este estudo oferece ao mundo científico é a seguinte: o tempo de RS é um parâmetro poderoso que pode ser utilizado na classificação de risco em pacientes com HAP. No entanto, são necessários ensaios clínicos multicêntricos, prospectivos e randomizados para melhor compreender a importância do tempo de RS em pacientes com HAP.
