# Supplementary material for: Marcador Prognóstico Eletrocardiográfico na Hipertensão Arterial Pulmonar: Tempo de RS
Source: Arq Bras Cardiol. 2024 Aug 21;121(9):e20240083. [Article in Portuguese] doi: 10.36660/abc.20240083 (PMC11495640; doi:10.36660/abc.20240083)
Supplement: Translation

# Translation: Electrocardiographic Prognostic Marker in Pulmonary Arterial Hypertension: RS Time

**DOI:** 10.36660/abc.20240083i

**Authors:** Emin Koyun, Anil Sahin, Ahmet Yilmaz, Ferhat Dindas, Idris Bugra Cerik, Gorkem Berna Koyun

**Affiliations:** 1 Sivas Turkey Sivas Numune Hospital, Department of Cardiology, Sivas – Turkey; 2 Sivas Turkey Sivas Cumhuriyet University Hospital, Department of Cardiology, Sivas – Turkey; 3 Usak Turkey Usak University Training and Research Hospital, Department of Cardiology, Usak – Turkey; 4 Ordu Turkey Ordu University Training and Research Hospital, Department of Cardiology, Ordu – Turkey; 5 Sivas Cumhuriyet University Hospital Sivas Turkey Sivas Cumhuriyet University Hospital, Department of Chest Disease, Sivas – Turkey

**Keywords:** Electrocardiography, Prognosis, Pulmonary Hypertension

## Abstract

**Background:**

Pulmonary hypertension is a condition that involves the remodeling of the right ventricle. Ongoing remodeling is also associated with disease prognosis. During the restructuring process, complex changes such as hypertrophy and dilatation may also be reflected in electrocardiographic parameters.

**Objectives:**

Our study aimed to investigate the relationship between prognosis and electrocardiographic parameters in patients with pulmonary arterial hypertension.

**Methods:**

The study was designed retrospectively and included patients diagnosed with pulmonary arterial hypertension between 2010 and 2022. The patients were divided into two groups based on their survival outcome. Various parameters, including electrocardiographic, demographic, echocardiographic, catheter, and blood parameters, were compared between the two groups. A p-value of <0.05 was considered statistically significant.

**Results:**

In the multivariate Cox analyses, the parameters that were found to be independently associated with survival were the 6-minute walk test, mean pulmonary artery pressure, presence of pericardial effusion, and time between the beginning of the QRS and the peak of the S wave (RS time) (p<0.05 for each). Of all the parameters, RS time demonstrated the best diagnostic performance (AUC:0.832). In the survival analysis, a significant correlation was found between RS time and survival when using a cut-off value of 59.5 ms (HR: 0.06 [0.02-0.17], p < 0.001).

**Conclusions:**

According to the results of our study, a longer RS time is associated with poor prognosis in patients with pulmonary arterial hypertension. We can obtain information about the course of the disease with a simple, non-invasive parameter.

## Introduction

Pulmonary hypertension (PH) is a rare, complex, chronic, and progressive disease resulting from a wide variety of underlying conditions that directly or indirectly lead to high pressures in the pulmonary arteries. PH is a hemodynamic and pathophysiological term that encompasses a diverse range of diseases affecting the cardiac and respiratory systems, with symptoms dependent on the affected system. Its hemodynamic definition is mean pulmonary artery pressure (mPAP) measured >20 mmHg at rest on right heart catheterization (RHC).^1^ Pulmonary arterial hypertension (PAH) is a group of PH that is diagnosed by excluding other precapillary causes, such as chronic thromboembolic pulmonary hypertension and PH due to lung disease. In this group, RHC parameters should be as follows: mPAP>20 mmHg, pulmonary capillary wedge pressure (PCWP) ≤15 mmHg, and pulmonary vascular resistance (PVR)>2 wood units (WU).^2^

The current treatment algorithm for patients with PAH requires frequent evaluation of the patient about prognosis and escalation of therapy if low-risk status is not reached. Physical examination for risk assessment, cardiopulmonary exercise test, World Health Organization functional class (WHO-FC), N-terminal pro-Brain type natriuretic peptide (NT-proBNP) level, 6-minute walk test (6-MWT), imaging studies and various diagnostic parameters, including RHC have been proposed.^3^

To date, there are limited studies on the role of electrocardiogram (ECG) in monitoring PAH patients. In PAH, changes such as dilatation and hypertrophy occur in the right ventricle (RV) over time due to high pulmonary artery pressure. These changes may also be reflected in patients’ surface ECGs. Although hemodynamic parameters and right ventricular hypertrophy have been shown to cause significant changes in the ECG,^4^ their prognostic value has not been extensively evaluated.

In a recent study on acute pulmonary embolism, a disease that affects the right heart, it was found that time between the beginning of the QRS and the peak of the S wave (RS time) is associated with mortality.^5^ However, it has not been investigated whether the RS time is related to mortality in specific PH subgroups, especially in chronic conditions. It is not yet clear whether ECG parameters at the time of diagnosis provide information about the course of the disease and survival in PH patients. In PAH, there is a delay in the intracardiac conduction system, especially due to the increase in pressure in the right heart chambers. As a result of the electrical conduction delay, stretching occurs in the S wave.^5^ As a result, it causes the time between the RS time to increase.^5^ Therefore, we aimed to investigate the relationship between ECG patterns of PAH patients at the time of diagnosis, especially the duration of RS, and the prognosis of the disease.

## Methods

All patients who underwent RHC at a tertiary hospital between 2010 and 2022 and were diagnosed with group 1 PAH were included in the retrospective study. The total number of patients with PAH who met these criteria was determined as 143. Then, for convenience, 143 healthy volunteers, matched in terms of age and gender and equal to the number of the patient group, were included in the study as a control group. In the first step, demographic characteristics, ECG, blood parameters, and echocardiographic parameters were compared between patients diagnosed with PAH and the control group of healthy volunteers. Then, PAH patients were divided into two groups: living and dead. Demographic characteristics, ECG, RHC, echocardiography, and blood parameters were compared between surviving and deceased patients (Central Illustration).

All the PAH patients included in the study had a medium or high risk for PH on transthoracic echocardiography. Therefore, these patients underwent RHC. The inclusion criteria for the study are as follows: Diagnosis of group 1 PAH according to the clinical classification in the PH guideline created by the European Cardiology and European Respiratory Society in 2022; RHC and mPAP>20 mmHg, PCWP≤15, PVR>2 WU; Being over 18 years old; presence of ECG at the time of diagnosis.

The exclusion criteria of the study are as follows: Being diagnosed with PH due to left heart disease; diagnosis of PH associated with lung diseases and hypoxia; Diagnosis of PH due to pulmonary artery occlusions; being diagnosed with PD with unclear and multifactorial mechanisms; Being under 18 years of age; missing data on RHC parameters at diagnosis; absence of ECG at diagnosis; if you have severe kidney or liver failure; having a history of malignancy.

ECGs of the patients at the time of diagnosis were found in the patient files. ECGs taken when the patients were diagnosed with PH were included in the study. ECGs with 12 leads, paper speed 25 mm/s, and 10 mm/mV were digitally scanned and uploaded to the software program on the computer, and all measurements were made with the software program on the computer. Two cardiologists performed the ECG examinations using this software program. If problems were detected in the ECG examinations, support was received from a third cardiologist. The cardiologists who reviewed the ECGs were blind to the patients’ results. On ECG; atrial arrhythmia, QT time, PR time, T wave inversion, QRS duration, right axis deviation, ST depression, RS time, and heart rate per minute were analyzed in a computer environment. QRS time; It was measured from the lead where the time between the beginning of the QRS wave and the J point was highest. In patients with PH, conduction delay and right and posteriorly orientation of the QRS vector occur due to hemodynamic changes. Accordingly, a prolongation of the RS duration occurs in the inferolateral leads on the ECG. Therefore, we calculated the RS duration in the patients’ ECG from the inferolateral leads. RS time was calculated from the lead with the highest RS time among the inferolateral leads (D1, D2, D3, AVL, AVF, V4, V5, and V6).^5^ The time between the starting point of the QRS wave and the lowest point of the S or S’ wave was determined as the RS time^5^ (Figure 1). The unit of measurement was determined as milliseconds.

**Figure 1:** Calculation of RS time on patients’ ECGs, ECG examples showing increased RS time (A) and normal RS time (B).

### Statistical analysis

The Kolmogorov-Smirnov test was used to determine the normal distribution of the data. Mann-Whitney U test or unpaired Student T-test was used to detect differences in continuous variable data. Continuous data were expressed as mean ± standard deviation (SD) or median and interquartile range. Categorical variables were expressed as absolute (n) and relative frequencies (%) Chi-square analysis was used to compare categorical variables. Univariate and multivariate Cox regression analysis was performed to determine parameters predicting mortality. Statistically significant variables (p<0.05) in univariate Cox regression analysis were included in multivariate Cox regression analysis. Receiver operating characteristic (ROC) curve analysis was performed to obtain the cutoff value and area under the curve (AUC) of parameters predicting mortality. Using Pearson Correlation analysis, the relationship between RS time and right heart functions on echocardiography was examined. Kaplan-Meier analysis was performed to examine the relationship between RS time, which has the highest diagnostic performance according to Roc curve analysis, and survival. The data were analyzed with the SPSS 22.0 statistical program. It was stated that p<0.05 was required for the data to be considered statistically significant.

## Results

When demographic, laboratory, echocardiographic, and ECG parameters of PAH (n=143) and the control group (n=143) were compared, RS duration, QRS duration, right axis deviation, atrial arrhythmia, ST depression, T wave inversion was higher in the PAH group than in the control group and was statistically significant. A detailed comparison of parameters between groups is shown in Table 1.

**Table 1:** Comparison of variables between the pulmonary arterial hypertension group and the control group

Variables		PAH group (n=143)	Control group (n=143)	p-value
Age (years)		62.32±13.43	59.79±14.45	0.12
Male n (%)		36 (25.17)	42 (29.37)	0.42
Body mass index (kg/m^2^)		28.74±1.81	26.53± 1.74	0.09
Echocardiographic parameters				
	Left ventricular ejection fraction (%)	55.15±3.26	56.49±3.13	0.37
	Left atrium diameter (mm)	40.1±5.3	36.4±3.3	0.08
	Interventricular septum (mm)	10.4±1.3	10.1±1.4	0.13
Blood parameters				
	Hemoglobin (g/dL)	13.7±2.1	14±1.9	0.29
	White blood cell (10^3^ /uL)	8.1±2.87	8.3±2.89	0.52
	Platelet (10^3^ /uL)	231.3±70.1	240.8±76.9	0.27
	Sodium (mEq/L)	137.8±3.7	138.2±3.2	0.31
	Potassium (mEq/L)	4.37±0.47	4.42±0.48	0.43
	Calcium (mg/dL)	8.92±0.55	8.89±0.55	0.73
	Creatinine (mg/dL)	0.83±0.24	0.80±0.19	0.29
	Aspartate aminotransferase (IU/L)	21.2±11.3	22.2±10.4	0.45
	Alanine aminotransferase (IU/L)	18±15.5	21.2±19	0.12
ECG parameters				
	QT time (ms)	402±5	403±12	0.14
	PR time (ms)	142.7±16	143.4±11.5	0.66
	Heart rate per minute	82.8±14.5	81±15.3	0.36
	RS time (ms)	60.7±10.2	55±4.3	<0.001
	QRS time (ms)	100.2±14.6	96.8±13.6	0.04
	Right axis deviation, n (%)	22 (15.38)	7 (4.8)	0.002
	ST depression, n (%)	25 (17.4)	3 (2.09)	<0.001
	Atrial arrhythmia, n (%)	27 (18.88)	0 (0)	<0.001
	T wave inversion, n (%)	21 (14.68)	6 (4.19)	<0.001

Data are expressed as n(%), mean ± standard deviation. PAH: pulmonary arterial hipertension. ECG: electrocardiogram.

PAH patients were divided into two groups: Surviving patients (n=108) and dead patients (n=35). Demographic characteristics, comorbidities, treatments used, 6-MWT, WHO-FC classification, laboratory, echocardiographic, ECG, and RHC parameters were compared between the two groups. In the deceased patient group, 6-MWT was less and found to be statistically significant. WHO-FC 3-4, mPAP, pericardial effusion, tricuspid jet velocity, RS time, and QRS time were higher in the deceased group and were found to be statistically significant. Detailed comparison between deceased and surviving patient groups in PAH patients is shown in Table 2.

**Table 2:** Comparison of demographic, echocardiographic, electrocardiographic, right heart catheterization, and laboratory parameters of deceased and surviving pulmonary arterial hypertension patients

Variables		Dead (35)	Survive (108)	p-value
Age (years)		64.45±10.6	61.63±14.1	0.21
Male, n (%)		10 (28.57)	26(24.07)	0.59
Body mass index(kg/m^2^)		28.4±1.9	28.8±1.7	0.24
6-minute walk test (m)		210.8±92.4	261.5±97.3	0.012
WHO-FC 3-4, n (%)		14 (40)	25 (23.14)	0.036
Diabetes mellitus, n (%)		7 (20)	21(19.44)	0.9
Hypertension, n (%)		9 (25.7)	28(25.92)	0.9
Coronay artery disease,n (%)		3 (8.57)	5(4.62)	0.38
**Right heart catheterization parameters**				
	sPAP (mmHg)	64.3±17.2	55.5±15.1	0.06
	dPAP (mmHg)	34.4±9.3	26.2±7.6	0.09
	mPAP (mmHg)	44.2±15.8	36.1±11.4	0.007
	Qp/Qs	1.04±0.20	1.04±0.13	0.82
	Vasoreactivity, n (%)	2 (5.7)	7 (6.5)	0.87
	PVR (WU)	7.05±3.2	7.18±2.6	0.80
	SVR (WU)	20.1±7	20.5±5	0.68
	PVR/SVR	0.357±0.09	0.359±0.11	0.90
	Cardiac index (L/min/m^2^)	2.79±0.44	2.81±0.46	0.81
	Partial oxygen pressure (%)	48.59±9.1	48.22±8.9	0.83
	RAP (mmHg)	8.70±1.32	8.76±1.71	0.84
	Cardiac output (L/min)	4.75±0.78	4.63±0.83	0.46
	PCWP (mmHg)	11.2±3.1	10.5±2.9	0.24
	Aortic systolic pressure (mmHg)	114.9±9.8	117.5±10.1	0.19
	Aortic diastolic pressure (mmHg)	65.2±12.7	69.1±12.3	0.11
**Echocardiographic parameters**				
	Left ventricular ejection fraction (%)	54.7±3.27	55.2±3.26	0.36
	Left atrium diameter (cm)	4.07±0.64	3.99±0.48	0.50
	Interventricular septum (cm)	1.06±0.17	1.03±0.12	0.31
	Right ventricle dilatation, n (%)	20 (57.14)	55 (50.92)	0.19
	Pericardial effusion,n (%)	13 (37.14)	5 (4.62)	<0.001
	Tricuspid jet velocity (m/s)	3.96(3.6-4.2)	3.72(3.4-4)	0.037
	sPAP (mmHg)	69±16.7	61±18.8	0.07
**Laboratory parameters**				
	Hemoglobin (g/dL)	13.9±2.3	13.6±2.0	0.56
	Platelet (10^3^ /uL)	229.1±91.7	232±62.11	0.83
	Sodium (mEq/L)	137.2±3.06	138±3.97	0.28
	Potassium (mEq/L)	4.43±0.55	4.36±0.44	0.45
	Calcium (mg/dL)	8.84±0.60	8.94±0.53	0.33
	Creatinine (mg/dL)	0.87±0.22	0.81±0.40	0.26
	NT-proBNP (pg/ml)	1491(0-2542)	1261(0-2180)	0.33
	High-density lipoprotein (mg/dL)	39.9±14.2	43±12.1	0.27
	Low-density lipoprotein (mg/dL)	110.6±30.3	102.6±36.8	0.26
	Triglyceride (mg/dL)	134.5±58.6	129.6±64.8	0.70
	Aspartate aminotransferase (IU/L)	19.4±16.6	21.8±9	0.42
	Alanine aminotransferase (IU/L)	17.3±15.4	18.2±15.6	0.77
**Medications**				
	Angiotensin-converting enzyme inhibitor, n (%)	10(28.57)	29 (26.85)	0.95
	Calcium channel blocker, n (%)	7(20)	21 (19.44)	0.23
	Betablocker, n (%)	9(25.71)	23 (21.29)	0.58
	Antiaggregant, n (%)	2(5.71)	6 (5.55)	0.52
	Anticoagulant, n (%)	9(25.71)	21 (19.44)	0.43
	Statin, n (%)	8(22.85)	25 (23.14)	0.26
	Endothelin receptor antagonist, n (%)	17(48.57)	59 (54.62)	0.50
	PDEi-5 inhibitors, n (%)	3(8.57)	10 (9.25)	0.51
	Riosiguat, n (%)	2(5.71)	8 (7.76)	0.46
	Prostanoid, n (%)	6(17.14)	19(17.59)	0.65
**Electrocardiographic parameters**				
	QT time (ms)	401.8±5.4	402.1±4.9	0.74
	PR time (ms)	141±16	143.2±16	0.49
	Heart rate per minute	84.3±15.4	82.2±142	0.47
	RS time (ms)	71.6±12.2	57.1±6.2	<0.001
	QRS time (ms)	107±12.8	98±14.4	0.001
	Right axis deviation, n (%)	5(14.28)	17(15.74)	0.38
	ST depression, n (%)	6(17.14)	19(17.59)	0.96
	Atrial arrhythmia, n (%)	7(20)	20(18.51)	0.85
	T wave inversion, n (%)	5(14.28)	16(14.81)	0.92

Data are expressed as n(%), mean ± standard deviation, and median (1st quartile - 3rd quartile). mPAP: mean pulmonary artery pressure; sPAP: pulmonary artery systolic pressure; dPAP: pulmonary artery dyastolic; NT-proBNP: terminal pro B-type natriuretic peptide; PCWP: pulmonary capillary wedge pressure; PDEi-5: phosphodiesterase type 5; PVR: pulmonary vascular resistance; RAP: right atrium pressure; SVR: systemic vascular resistance; WHO-FC: World Health Organization functional classification.

Univariate and multivariate Cox regression analysis was performed to identify predictors of mortality in PAH patients. In multivariate Cox regression analysis, 6-MWT, mPAP, presence of pericardial effusion, and RS time were found to be independent predictors of mortality in PAH patients (Table 3).

**Table 3:** Univariate and multivariate Cox regression analysis to identify predictors of mortality

Variables	Univariate regression analysis				Multivariate regression analysis			
	HR	CI		p	HR	CI		p
6-Minute walk test	0.995	0.990	0.999	0.006	0.991	0.985	0.997	0.003
WHO-FC 3-4	2.492	1.247	4.979	0.013				
mPAP	1.035	1.017	1.053	<0.001	1.059	1.029	1.090	<0.001
Pericardial effusion	4.878	2.438	9.761	<0.001	3.414	1.309	8.900	0.012
Tricuspid jet velocity	2.679	0.993	7.225	0.052				
RS time	1.182	1.139	1.228	<0.001	1.215	1.140	1.295	<0.001
QRS time	1.038	1.017	1.059	<0.001				

CI: confidence interval; HR: hazard ratio; mPAP: mean pulmonary artery pressure; WHO-FC: World Health Organization functional classification.

ROC analysis was performed to evaluate the diagnostic performance of RS time, which is an independent predictor of mortality, on prognosis. In the ROC analysis, the value of 59.5 ms was determined as the cut-off for the RS time. An RS duration greater than 59.5 ms; it was determined to predict mortality in PAH patients with 85.7% sensitivity and 79.6% specificity (Figure 2). According to ROC curve analysis, the cut-off value of a high sensitive RS time that can be used in clinical use can be determined as 56.5 ms (sensitivity 94%, specificity 54%), and the cut-off value of a high specificity RS time can be determined as 76.5 ms (sensitivity 44%, specificity 93%). According to the results of Roc curve analysis, we found that the strongest parameter predicting mortality was RS time [(6-MWT; AUC:0.658, p=0.008), (mPAP; AUC:0.674, p=0.004), (pericardial effusion; AUC:0.641, p=0.019)].

**Figure 2:** Evaluation of RS time with ROC analysis.

Using Pearson Correlation analysis, the relationship between RS time and right heart functions on echocardiography was examined. According to the analysis results, a significant and positive relationship was found between RS time and right ventricular dilatation (r=0.243, p<0.05), sPAP (r=0.265, p<0.05), and tricuspid jet velocity (r=0.652, p<0.05).

The median follow-up period of the deceased patient group after the diagnosis of PAH was determined as 30.4 months. In the Kaplan Meier survival analysis performed by setting the cut-off value for RS time as 59.5 ms, a statistically significant correlation was found between RS time and survival [HR:0.06(0.02-0.17), p<0.001)] (Figure 3).

**Figure 3:** Relationship between RS time and survival in Kaplan Meier analysis.

## Discussion

According to the results of our study, several parameters can provide information about the prognosis of PAH patients at the time of diagnosis. In addition to the predefined parameters, the RS time we can detect on the ECG can give us important information. Additionally, our study has shown that it can provide better prognostic information than many parameters evaluated at the time of diagnosis.

PAH is a disease characterized by a persistent, abnormal increase in pulmonary artery pressure. As a result, right ventricular failure develops, with clinical symptoms of shortness of breath, fatigue, weakness, angina, and syncope.^6^ Today, PAH continues to be a chronic disease whose pathogenesis is not fully elucidated.^7^ Detailed clinical evaluation of the patient plays a key role in the selection of treatment and the observation of the patient's response to treatment.^8^ Therapeutic decisions in PH should be based on parameters with proven prognostic value.^9^

While echocardiography is given priority in routine practice in screening for PH, ECG is an examination recommended to be used in diagnostic steps. However, despite a relatively low sensitivity and specificity, the ECG is still useful in the early stage of diagnosis of PH. In an ECG-based study conducted in Japanese school children, it was found that ECG could predict the diagnosis of PAH early.^10^ It has been shown that ECG used to screen for PH can provide significant benefit when combined with other non-invasive tests.^11^

Various ECG changes such as increased duration and amplitude of the P wave in D2, changes in the voltage and heart rate of the precordial leads, changes in QTc and QRS duration, and the presence of the qR wave in lead V1 affect the prognosis in PAH patients.^12^ Tonelli et al. compared ECGs of patients with PAH at diagnosis and the end stage of the disease. In this study, an increase in the QRS duration, PR duration, QTc duration, R/S amplitude ratio, and heart rate in lead V1 was observed in the ECGs taken at the last stage of the disease. In addition, negative T waves in the inferior leads, deviation to the right in the axis of the QRS complex, and right bundle branch block were observed more frequently.^13^ In our study, QRS duration was longer and T wave inversion was detected more frequently in the deceased patient group.

There are several studies on RS time. In a retrospective study examining the ECG parameters measured during the diagnosis of acute pulmonary embolism, the RS duration and one-month mortality of the patients were examined. One-month mortality of patients was found to be related to the longer duration of RS.^5^ However, while this study examined the relationship between RS duration and short-term mortality, our study examined the relationship between RS duration and longer-term mortality.

In our study, RS time, an electrocardiographic parameter, was found to be longer in the patient group who died during follow-up than in the survivors. It was determined that it could predict mortality in univariate and multivariate Cox regression analyses. Moreover, according to our literature review, our study is the first and largest study examining the relationship between PAH and RS time. Current guidelines recommend risk-stratifying patients with PAH at baseline and at each follow-up visit. This risk classification includes several parameters, including clinical, laboratory, and imaging methods. In addition to routine risk stratification parameters, measuring RS duration on ECG at diagnosis and during follow-up can strengthen prognostic information.

Conditions that affect the QRS complex also affect RS duration. Because RS time is part of QRS time. In PAH, the QRS wave being longer than normal is associated with hemodynamic changes. Increased right ventricular afterload causes dilatation and loss of function in the RV. This situation may affect the right bundle branch and Purkinje fibers, causing conduction delay or block.^14^ In PAH, conduction delay and right and posteriorly orientation of the QRS vector cause prolongation of the RS duration, especially in the inferolateral leads. In people without heart disease, the S wave in leads V4-V5-V6 is caused by the direction of right ventricular and septal electrical forces towards the heart base and the left ventricular electrical forces towards the back of the heart.^15^ Therefore, RS time in inferolateral leads may have predicted poor prognosis in PAH better than other ECG parameters.

Echocardiography has a very important role in the diagnosis of PAH. This is because it is readily available and non-invasive. In addition, many parameters measured by echocardiography have been shown to be related to pulmonary hemodynamics.^16^ Therefore, echocardiography can provide us with important hemodynamic parameters regarding the diagnosis, follow-up, and prognosis of PAH patients.^17^ In patients with PAH, increased pulmonary artery pressure causes hypertrophy and dilatation in the RV.^18,19^ As a result, the RV enlarges and may become larger than the left ventricle over time. Therefore, more right ventricular dilatation and poor clinical outcome have been detected in PAH patients.^20^ Following changes in the RV, right ventricular failure may develop in these patients. As a result of right ventricular failure, tricuspid valve insufficiency may begin, and an increase in the jet velocity in the tricuspid valve may occur. It is important to evaluate pericardial effusion echocardiographically in patients with PAH. Studies have found that PAH patients with pericardial effusion have poor clinical outcomes and a high mortality rate.^21^ When echocardiographic parameters were compared between deceased and living PAH patients, it was observed that tricuspid jet velocity and pericardial effusion rate were higher in the deceased patient group. However, according to the regression analysis results, it was found that there was a significant relationship only between pericardial effusion and mortality among the echocardiographic parameters. This was considered to be consistent with the current literature. Therefore, regular evaluation of the presence of pericardial effusion in PAH patients during the follow-up period is very important for its prognostic benefit.

Exercise capacity is associated with survival and functional status in PH patients.^22^ Exercise capacity assessed by the six-minute walk test has been a mandatory parameter in most recent clinical studies on PAH.^23^ Additionally, a study found that 6-MWT could also evaluate exercise-induced oxygen desaturation in patients with pulmonary vascular disease.^24^ However, few studies have been conducted to investigate the relationship between pulmonary function and effort-related desaturation in PH patients.^25^ In our study, the average 6-MWT was found to be lower in deceased PAH patients than in survivors. Additionally, univariate and multivariate Cox regression analyses concluded that there was a significant relationship between mortality and 6-MWT. From this perspective, it is thought that aiming to increase 6-MWT in the follow-up and treatment of PAH patients is a correct treatment target.

mPAP is an important parameter in the diagnosis of the disease.^26^ In our study, the mPAP measured by RHC was found to be statistically significantly higher in the deceased patient group. Additionally, regression analyses revealed a significant association between mPAP and mortality. Although current guidelines do not accept mPAP measured by RHC as a risk parameter in the treatment follow-up of PAH patients, our results suggest that targeting the decrease in mPAP may be important in the follow-up of the disease.

### Limitations

The main limitations of the study are its retrospective nature, single-center design, and small number of participants. In addition, not including PH groups other than group 1 PAH patients in the study is an important limitation. Not being able to perform RHC and cardiopulmonary exercise testing during follow-up can also be shown as limitations.

## Conclusions

The results obtained from our study show that there is a strong relationship between RS duration and mortality in PAH patients. The novel insight that this study offers to the scientific world is this: RS time is a powerful parameter that can be used in risk classification in PAH patients. However, multicenter, prospective, and randomized controlled trials are needed to better understand the importance of RS time in patients with PAH.
